# Comprehensive Analysis of Different Subtypes of Oxylipins to Determine a LC–MS/MS Approach in Clinical Research

**DOI:** 10.3390/metabo16010004

**Published:** 2025-12-22

**Authors:** Yurou Zhao, Zhengyu Fang, Zeyu Li, Yizhe Liu, Yang Bai, Xiaoqing Wang, Hongjun Yang, Na Guo

**Affiliations:** 1 Experimental Research Center, China Academy of Chinese Medical Sciences, Beijing 100700, China; zyrou00@163.com (Y.Z.); m18631440795@163.com (Z.F.); chestnutfish1998@126.com (Z.L.); chloelyz2024@163.com (Y.L.); baiyang020421@163.com (Y.B.); Xiaoqing20502@163.com (X.W.); 2 School of Pharmacy, Shenyang Pharmaceutical University, Shenyang 110016, China

**Keywords:** oxylipin, extraction protocol, extraction recovery rate, matrix effect, clinical protocols

## Abstract

Background/Objectives: Different oxylipin subtypes have unique biological properties, requiring effective analytical protocols. However, establishing a complete pathway detection protocol for comprehensive oxylipin analysis is challenging. This study aimed to evaluate the adaptability and specificity of oxylipin subtypes under different extraction schemes and to develop a robust analytical platform for clinical biomarker investigation. Methods: We revealed the adaptability and specificity of oxylipin subtypes based on different single-step extraction schemes. A high-throughput quantitative automated solid-phase extraction coupled with a liquid chromatography–tandem mass spectrometry (aSPE–LC–MS/MS) analytical platform was established for a broad panel of complex oxylipins. The method was applied to serum samples of patients with coronary heart disease (CHD). Results: Our results verified that oxo-oxylipins, resolvin, and eicosanoids showed the best extraction efficiency under SPE protocol. Most hydroxy-oxylipins, dihydroxy-oxylipins, and HOTrEs are suitable for methanol protocol, HDHA for acetonitrile protocol, and epoxy-oxylipins for the methyl tert-butyl ether protocol, while medium-chain HETE is suitable for ethyl acetate protocol. Importantly, a novel sensitive fast method with wide coverage by the aSPE–LC–MS/MS analytical platform with satisfying sensitivity, accuracy and precision, extraction efficiency, low matrix effect, and linear calibration curves was obtained. Furthermore, we have successfully applied this method and found that 5-HETE, 11-HETE, and 15-HETE can serve as integrated biomarkers for patients with CHD, with high diagnostic performance. Conclusions: The study provides the best protocol for the clinically targeted detection of oxylipins and provides an important means for studying biomarkers of diseases.

## 1. Introduction

Oxylipins represent a class of bioactive lipid metabolites derived from polyunsaturated fatty acid (PUFA) precursors, specifically arachidonic acid (AA), linoleic acid (LA), α-linolenic acid (ALA), eicosapentaenoic acid (EPA), and docosahexaenoic acid (DHA) [1,2]. These compounds can be catalyzed by either the cyclooxygenase (COX), lipoxygenase (LOX), and cytochrome P450 (CYP450) enzymes, or a series of oxidative metabolites generated by automatic oxidation [3]. Oxylipins serve as crucial mediators in multiple physiological processes, including inflammation, immunity, endocrine function, and oxidative balance. Due to their bioactive roles in vivo, several studies have explored the role of oxylipins as potential biomarkers of diseases such as gout [4], cardiovascular diseases [5,6,7,8], obesity [9], and diabetes mellitus type 2 [10]. Therefore, it is crucial to be able to classify and thoroughly investigate the clinical significance of different kinds of oxylipins in many biological samples and obtain an integrated understanding of their chemical and biological complexity through high-throughput targeted metabolomics.

Recent advances in analytical technology have significantly enhanced oxylipin detection capabilities, particularly through sensitive liquid chromatography–mass spectrometry (LC–MS) platforms. For targeted oxylipins, ultra-performance liquid chromatography (UPLC) coupled with electrospray ionization (ESI) triple quadrupole tandem MS (MS/MS) is the preferred method [11]. The analysis of oxylipins based on LC–MS can reliably identify single oxylipins species and effectively separate isomers and isobaric compounds [12,13], improving the ability to comprehensively study multiple oxylipins in biological samples. A major challenge in oxylipin research stems from their structural diversity arising from multiple oxidative pathways. Different oxidation mechanisms generate numerous structurally similar oxylipin derivatives with comparable physicochemical characteristics [14,15,16]. In addition, most of the oxylipins are present in low concentrations and are easily affected by temperature, light, and residual enzymes in biological liquids [17]. Therefore, it is necessary to select the optimal pre-treatment methods for different kinds of oxylipins to achieve the targeted quantitative analysis of oxylipins.

Currently, the reported extraction schemes of oxylipins mainly include protein precipitation (PPT) [18], liquid–liquid extraction (LLE) [19], and solid-phase extraction (SPE) [19,20]. Cold organic solvents (−20 °C) are usually selected to destroy the noncovalent bonds between proteins and oxylipins, enabling the efficient extraction of oxylipins. Compared with other solvents, methanol (MeOH) has weaker hydrophobicity and higher extraction efficiency for oxylipin in serum [21]. In addition, acetonitrile (ACN) is often used as a solvent for the PPT of oxylipins in biological samples such as serum, plasma, and saliva [22]; however, the obtained extraction results are different from those obtained using extraction with MeOH. This may be due to the effect of ACN on the enzyme activity [23]. Furthermore, to improve the signal-to-noise ratio in the LC–MS analysis, it is necessary to further separate the oxylipins from the matrix components that may interfere with the LC–MS analysis by using either LLE or SPE. These steps not only remove the co-eluting matrix components but also concentrate oxylipins. LLE represents a prevalent sample preparation approach, particularly effective for lipid extraction encompassing phospholipids, ceramides, sphingomyelins, and cholesterol esters [24]. Among the numerous LLE methods, Folch’s method [25] and the Bligh and Dyer method [26] are highly praised for their efficiency and reliability. However, due to the potential toxicity risk of chloroform, the application of these two traditional methods in LC–MS analysis is significantly limited. Methyl tert-butyl ether (MTBE), as another primary lipid extraction and separation method, despite the limited literature reports on its extraction of oxylipins, has been successfully utilized by Rund et al. to analyze IsoP and IsoF formed in HCT116 cells [27]. Furthermore, studies have also shown the potential advantages of ethyl acetate (EA) LLE in the extraction of oxylipins. SPE has gained widespread adoption as a sample preparation technique owing to its superior recovery rates, reduced organic solvent consumption, and operational simplicity, especially for polar eicosanoids including LTB4, PGD2, PGE2, and PGF2α. Unfortunately, SPE kits are costly, and their use is time-consuming, which for unstable eicosanoids means that the longer extraction process hinders accurate analysis. Although PPT, SPE, and LLE are all commonly employed extraction techniques, they operate through distinct mechanisms and demonstrate varying recovery efficiencies. For oxylipin metabolomics research, comprehensive investigations comparing these methods’ performance across different oxylipin classes remain limited.

**Figure 1:**
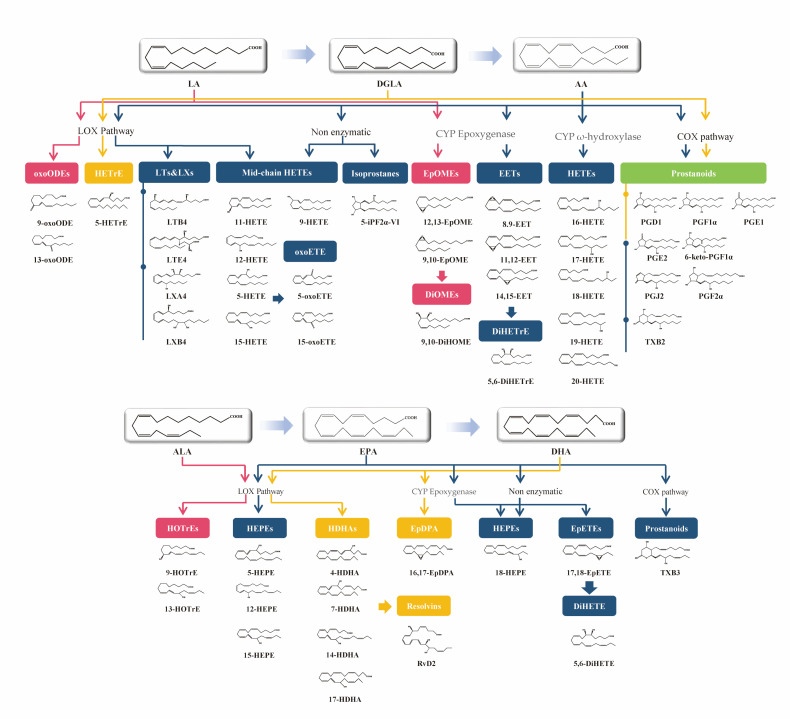
LA-, DGLA-, AA-, ALA-, EPA-, and DHA-derived oxylipins and the main enzymes involved in their generation and breakdown. Arrows of different colors represent distinct pathways.

The goal of this work is to propose the adaptability and specificity of oxylipin subtypes based on different single-step extraction schemes and establish a high-throughput quantitative scheme of oxylipins for serum. Firstly, five analytical methods of oxylipin subtypes were investigated, namely the MeOH PPT, ACN PPT, EA LLE, MTBE LLE, and SPE methods. Three key parameters—extraction efficiency, recovery rate, and matrix effects (MEs)—were assessed to identify the most suitable analytical approach for various oxylipin subclasses. For the further development of SPE methods, the performance of manual SPE (mSPE) and automated SPE (aSPE) methods were compared to establish a high-throughput quantitative oxylipins analytical platform of clinical serum. Eventually, a novel sensitive fast aSPE–LC–MS/MS analytical platform was applied to detect serum samples of coronary heart disease (CHD) patients to screen potential oxylipins biomarkers for the diagnosis and treatment of CHD (Figure 1).

## 2. Materials and Methods

### 2.1. Materials, Instruments, and Methods

The following LC–MS grade solvents were purchased from Fisher Scientific (Nidderau, Germany): ACN, formic acid (FA), isopropanol (IPA), and MeOH. Ultra-purity water (resistivity 18.2 MΩ·cm) was generated using a Milli-Q purification system (Bedford, MA, USA). Reference standards of oxylipins and corresponding internal standards (see Supplementary Table S1) were acquired from Cayman Chemicals (Ann Arbor, MI, USA). The high-throughput fully aSPE instrument was obtained from Reeko (RayKol Group Corp., Ltd. Xiamen, China).

### 2.2. Biological Samples

Serum specimens were obtained from two sources: Xiyuan Hospital affiliated with the China Academy of Chinese Medical Sciences, and male Sprague-Dawley rats (aged 6–8 weeks, average weight 240 g) supplied by Huafukang Biological Technology Co., Ltd. (Beijing, China). The study was designed as a methodological development and validation experiment. Blank rat serum samples were used as the biological matrix for assay optimization. No experimental or control groups were compared, as the study focused solely on establishing analytical performance parameters rather than evaluating interventional effects. The experimental unit was defined as an individual serum aliquot. Blood was collected in silica-coated tubes and left to coagulate vertically for 30–60 min at ambient temperature. Subsequently, the samples were centrifuged (3000× *g*, 10 min, 4 °C) to separate the serum. For method optimization, 50 μL aliquots of each processed serum sample were prepared and flash-frozen at −80 °C until further analysis.

All animal studies were performed following the guidelines of the Beijing Municipal Ethics Committee for the care and use of laboratory animals and were approved by the Animal Care & Welfare Committee, Chinese Academy of Medical Sciences (IRM-DWLL-2021114) (Beijing, China). The Ethics Committee of the Medical Experimental Center of the Chinese Academy of Chinese Medical Sciences approved the collection of samples, and written informed consent was obtained from each subject. All patient studies were approved by the ethics committee of the Medical Experimental Center of the Chinese Academy of Chinese Medical Sciences (WJEC-KT-2021-032-P003) and was conducted under the restriction of the ethical guidelines of the 1975 Declaration of Helsinki. Written informed consent was obtained from all participants included in the study.

A total of six blank rat serum samples were used for the methodological validation experiments. This sample size was selected based on common practice for preliminary assay development and validation, where six samples are typically sufficient to assess key parameters such as precision and matrix effects. All serum samples were blank samples obtained from healthy rats. No specific inclusion or exclusion criteria were applied, as all available samples were used for method validation.

### 2.3. Preparation of Biological Samples

To assess the efficiency of different extraction approaches for serum oxylipin analysis, six distinct protocols were systematically evaluated in parallel: two PPT techniques (using MeOH and ACN), two LLE approaches (with MTBE and EA), and two SPE methods (manual and automated processing). Each extraction method was performed in triplicate for comparative analysis. The sample treatment procedures are summarized in Figure S1. The ISs solution of 100 ng/mL included TXB2-D4, PGF2a-D4, RvD2-D5, LXA4-D5, LTE4-D5, 15-HEPE-D5, 17-HDHA-D5, 9-OxoODE-D3, and 12-HETE-D8. All the extraction solvents were cooled down in the 4 °C refrigerator prior to oxylipins extraction. All extraction protocols were compared by evaluating the abundance extraction efficiency, extraction recovery, and ME.

#### 2.3.1. PPT Method

The sample preparation protocol was standardized for both conditions. First, 2 μL of internal standard solution (100 ng/mL) was spiked into 50 μL of serum and thoroughly mixed. PPT was then performed by adding 150 μL of either MeOH or ACN. After vigorous vortexing, samples were chilled at −20 °C for 20 min followed by centrifugation (13,225× *g*, 5 min, 4 °C). The resulting supernatant was evaporated to dryness using nitrogen gas, then redissolved in 50 μL MeOH with 2 min of vortex mixing. A final centrifugation step (13,225× *g*, 5 min, 4 °C) was conducted prior to transferring the processed samples to vials for UPLC–MS/MS (liquid chromatography/mass spectrometry) analysis.

#### 2.3.2. LLE Method

In the LLE method, extractions were carried out with cold MTBE and cold EA. The specific procedure is as follows: take 50 μL of serum sample (including 2 μL ISs, with each IS concentration of 100 ng/mL) mixed with cold MTBE or EA (150 μL), and vortexed for 1 min. Following 20 min of −20 °C incubation, samples underwent centrifugation (13,225× *g*, 5 min, 4 °C). The resulting supernatant was carefully transferred to pre-chilled 1.5 mL Eppendorf tubes, then evaporated to complete dryness using nitrogen gas. The reconstitution process of the dried sample was the same as that for the PPT method and then the sample was used for UPLC–MS/MS analysis.

#### 2.3.3. SPE Method

Two SPE sample preparation procedures were performed in our study. The mSPE protocol was based on a manual Visiprep™ SPE device, and the aSPE protocol adopts the Fotector Plus automatic SPE equipment (RayKol Group Corp., Ltd.) controlled by the RILUTION LH software (version 60.0). Oasis HLB (hydrophilic–lipophilic balance) cartridges (1 cm^3^, 30 mg, Waters Corp.) were tested as stationary phases. Prior to SPE execution, all system components (columns, needles, valves) underwent aqueous flushing, followed by cartridge conditioning with sequential 2 mL MeOH and 2 mL Milli-Q water washes. A precise 2 μL aliquot of IS solution (100 ng/mL concentration) was introduced to 50 μL serum samples with thorough mixing. Following loading, the cartridges were washed with 10% MeOH (*v*/*v*, 2 mL). Then, vacuum (mSPE) or N_2_ (aSPE) treatment was performed for 10 min. The oxylipins were eluted with MeOH (1.5 mL). After drying under N_2_ gas, the samples were re-dissolved with MeOH (50 μL), vortexed for 2 min, and centrifuged for 5 min at 4 °C and 13,225× *g* for UPLC–MS/MS.

#### 2.3.4. Optimization of aSPE Extraction Efficiency

To optimize the extraction efficiency of aSPE, different washing, elution, and reconstitution conditions were evaluated and optimized, as described in Supplement Section S1. The optimized aSPE protocol was implemented as follows: an Oasis HLB cartridge was first preconditioned with 2 mL MeOH followed by 2 mL ultrapure water. A 50 μL serum aliquot was then loaded onto the prepared cartridge. After sample loading, the sorbent bed was rinsed with 2 mL water to remove impurities, followed by a 10 min nitrogen drying step to eliminate residual moisture. Finally, analytes were collected by eluting with 1.5 mL MeOH containing 0.02% FA into pre-labeled Eppendorf tubes. The eluate was evaporated to dryness under a stream of N_2_ and dissolved with 50 μL 50% ACN (*v*/*v*), centrifuged at 13,225× *g* for 5 min at 4 °C, and 5 μL of the final sample was injected into the LC–MS instrument for analysis. The oxylipins extraction protocol optimized workflow is presented in Figure S2.

### 2.4. UPLC–MS/MS Analysis

The analytical platform comprised a Waters ACQUITY UPLC system coupled to a Xevo TQ-S triple quadrupole mass spectrometer (Waters, Milford, MA, USA). Separation was achieved on a BEH C18 column (2.1 × 100 mm, 1.7 μm; Waters) maintained at 40 °C. The mobile phase system was employed: Phase A: ACN/water (45:55, *v*/*v*) with 0.02% FA; Phase B: ACN/IPA (50:50, *v*/*v*); a binary gradient program was executed at 0.4 mL/min: 0–50% B over 10 min, with 5 μL injection volume. The autosampler washing protocol utilized was as follows: strong wash: ACN/water (90:10, *v*/*v*); weak wash: ACN/water (10:90, *v*/*v*).

Analysis was performed in negative ionization mode with multiple reaction monitoring (MRM) detection. Key ESI conditions included the following: capillary voltage: 2 kV, source temperature: 100 °C, desolvation temperature: 400 °C, desolvation gas (N₂) flow rate: 800 L/h, cone: 150 L/h. All data were acquired using Masslynx 4.1 software (Waters) and processed with TargetLynx application. See Table S2 for more details on MRM methods.

### 2.5. Method Validation Parameters

#### 2.5.1. Reproducibility and Recovery

The 50 component standards used for preparing standard quality control (SQC) samples were shown in Table S1. The concentration of all standards was 25 ng/mL. System reproducibility was determined by calculating coefficient of variation (CV) values for both internal standards and SQC samples. Method precision between manual and aSPE approaches was evaluated through CV distribution analysis of feature intensities across triplicate runs for each protocol.

Recovery rates served as additional metrics for extraction performance comparison. These were determined by the following: Pre-extraction spiking: Adding 2 μL of standard solution (25 ng/mL for each analyte) prior to sample processing; Post-extraction spiking: Adding equivalent standards after protocol completion. The recovery percentage was then calculated by comparing the respective peak areas from these two approaches.

#### 2.5.2. Matrix Effect

The matrix effect (i.e., the suppression or enhancement of the metabolite signal) was evaluated by analyzing the peak area of all oxylipins prepared in the solvent matrix (MeOH) and the extracted serum matrix. Post-extraction spiked sample was prepared by spiking 2 μL SQC into the serum after the sample preparation protocols. The ME can be calculated as follows:

ME % = [(Peak Area Post Extraction Spiked − Peak Area Post Extraction Unspiked)/Peak Area standard] × 100%

#### 2.5.3. Linearity, Accuracy, Precision, and Sensitivity

The developed method was suitably validated for linearity, precision, accuracy, and sensitivity to meet the needs of our clinical application. Calibration curves were generated by plotting analyte-to-internal standard peak area ratios versus nominal concentrations. Linearity was assessed through determination coefficients (r^2^) derived from regression analysis. Method accuracy was evaluated by quantifying the agreement between measured concentrations (calculated from calibration curves) and known spiked concentrations using the following formula:

Accuracy = Calculated conc. from calibration curve/Spiked conc. × 100%

Method precision was evaluated by calculating the percentage CV (%CV) from triplicate measurements. The assay’s sensitivity was characterized by determining both the detection limit (LOD) and quantification limit (LOQ), defined as the minimum analyte concentrations yielding signal-to-noise ratios of 3:1 and 10:1, respectively. Both LOD and LOQ were estimated by visual inspection of the chromatogram signal-to-noise ratio at different concentrations of analytes.

### 2.6. Application to CHD Clinic Serum Sample Oxylipins Metabolomics Analysis

This study enrolled 30 CHD patients of 48–75 years of age from Xiyuan Hospital at the China Academy of Chinese Medical Sciences, as well as control participants free of coronary artery disease or atrial fibrillation. CHD diagnosis was confirmed through either conventional angiography or CT angiography, with documented stenosis exceeding 50% in one or more major coronary arteries. Detailed patient selection criteria, including inclusion/exclusion parameters, are available in Supplementary Section S2. Following the sample processing protocol outlined in Section 2.2, all serum specimens underwent UPLC–MS/MS analysis. The baseline participant characteristics are summarized in Table S3, and the standard curves utilized for the major oxylipin concentrations are described in Table S5.

### 2.7. Statistics Analysis

The analytical data were processed using SIMCA-P (v14.1) to conduct partial least squares discriminant analysis (PLS-DA), enabling differentiation between the health control (HC) and CHD cohorts. Model validation was achieved through 200 permutation tests to ensure statistical reliability. Metabolite data have a normal distribution by Shapiro–Wilk test and Quantile–Quantile plots. Independent samples *t*-tests and receiver operating characteristic (ROC) curve analyses were performed using GraphPad Prism (version 8.0; GraphPad Software, Inc., San Diego, CA, USA). A *p*-value < 0.05 corrected by Bonferroni and fold change (FC) < 0.667 or >1.5 was used as the cutoff for the significance of differential metabolites. Column diagrams were drawn by GraphPad Prism 8.0 (GraphPad Software Inc., USA). Furthermore, the potential biomarkers were evaluated by the ROC analysis. The AUC (area under the curve) was used to assess the performance of the model.

## 3. Results

### 3.1. Number and Overview of Extracted Oxylipins

**Figure 2:**
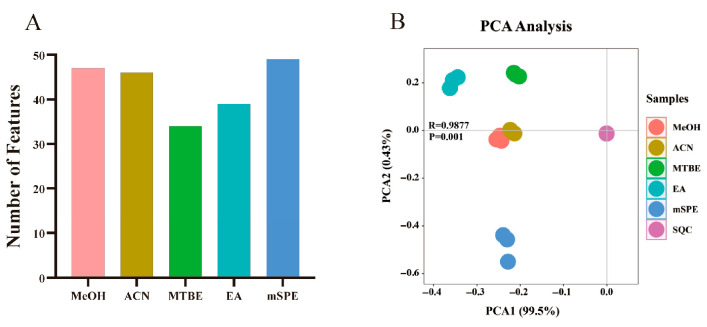
Evaluation of five oxylipin extraction methods based on feature detection and metabolic profiling. (**A**) Bar graph showing the count of consistently detected oxylipins for each method. mSPE and PPT methods (MeOH, ACN) detected more features than LLE methods (MTBE, EA). (**B**) PCA score plot illustrating the metabolic variation introduced by different extraction protocols. The tight cluster of SQC samples confirms system stability. Distinct, method-dependent clustering is observed: PPT methods (MeOH, ACN) and LLE methods (MTBE, EA) form two separate, tight clusters, while the mSPE protocol separates from both and shows greater dispersion among its replicates.

The number of oxylipins consistently detected across triplicate analyses for each extraction method is shown in Figure 2A, revealing statistically significant differences in the detected features among the five protocols. Both mSPE and PPT methods yielded higher oxylipin counts than LLE techniques (Figure 2A). For initial data exploration, principal component analysis (PCA) score plots were generated from the complete dataset (containing biological and SQC samples) to examine protocol-induced metabolic variations and assess analytical repeatability (Figure 2B). The close clustering of SQC samples in PCA space demonstrated good system reproducibility during UPLC–MS/MS analysis. Furthermore, the score plots exhibited a clear separation between different extraction protocols (Figure 2B).

Analytical repeatability during UPLC–MS/MS operation was verified through sequential SQC sample injections for each dataset. The compact clustering pattern of SQCs in PCA score plots (Figure 2B) confirmed system reproducibility, while clear method-specific segregation was evident among the various extraction protocols. The replicates of the MeOH and ACN protocols were tightly clustered, indicating their similar efficiency and metabolomic profiles. The EA protocol extraction replicates were clustered close to the results for the MTBE protocol compared with the PPT protocols (MeOH and ACN), suggesting the difference in the types of extracted oxylipins between LLE and PPT. Previous studies indicate that EA extraction demonstrates comparable efficiency to MTBE while offering reduced toxicity, aligning with our experimental findings. This similarity in performance likely stems from their analogous molecular structures and consequent physicochemical characteristics. An evident separation was noted between the mSPE protocol extraction and the results of other protocols (Figure 2B). However, the clustering of the mSPE protocol extraction in the PCA score graph was scattered, indicating that SPE may have unsatisfactory protocol reproducibility. These normalized data clustered by different extraction protocols indicated the differences in the oxylipin profiles and extraction efficiencies of the different protocols.

### 3.2. Advantages of Different Extraction Methods for Extracted Oxylipins

Following the above-described investigation, we further investigated the advantageous extraction protocols of different oxylipins and examined the extraction efficiency, extraction recovery rate, and MEs of the protocols.

#### 3.2.1. Extraction Efficiency

**Figure 3:**
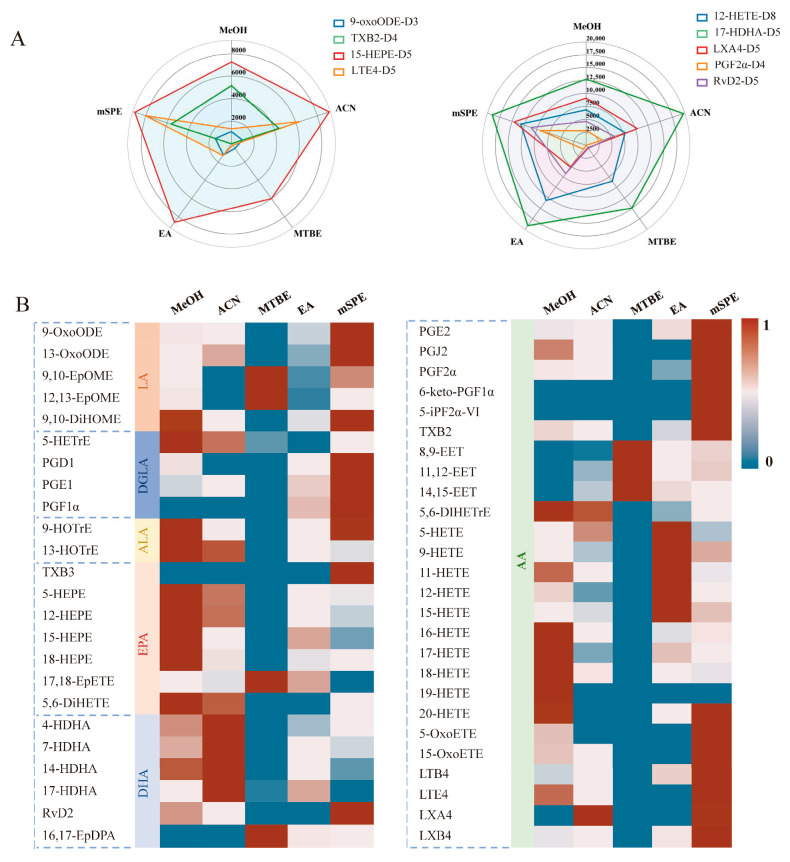
Comparison of extraction efficiency across different protocols. (**A**) Radar diagrams of peak area of IS extracted from different protocols MeOH, ACN, EA, MTBE, and mSPE. For each extraction method *n* = 3. (**B**) Heatmaps reflecting the peak area of standard unlabeled analytes from serum. “1” represents the maximum peak area in five protocols; “0” represents the minimum peak area in five protocols.

The extraction efficiency of various solvents was initially assessed by comparing IS peak areas. As illustrated in Figure 3A, the evaluation of five extraction methods revealed the superior performance of 12-HETE-D8 when using EA as the extraction solvent. The extraction result of 17-HDHA-D5 performs best under the extraction conditions of ACN, while the extraction result of 15-HEPE-D5 performs best under the extraction conditions of MeOH. By contrast, for LXA4-D5, PGF2 α-D4, and RvD2-D5, the best results were achieved with mSPE as the extraction protocol. The mSPE protocol also showed the best 9-oxoODE-D3 extraction efficiencies, while the ACN and SPE protocols can be considered as the best choice for TXB2-D4. For LTE4-D5, a significant advantage is observed for MeOH and mSPE.

These findings indicate that certain extraction methods are more suitable for extracting different classes of oxylipins. However, relying on a single or a small number of ISs may not be enough to capture all types of oxylipins and represent all relevant oxylipin species. To address this limitation, the average peak area of all oxylipins was used to evaluate the effectiveness of extraction protocols for the entire range of metabolized oxylipins, as illustrated in Figure 3B. Among these, oxylipins in the classes of oxoODE, oxoETE, PGs, iso-PGs, TXs, Rvs, LTs, and LXs showed the best extraction efficiencies under SPE conditions. HETrE, HEPE, ω-terminal HETE, DiHETE, DiHOME, DiHETrE, and HOTrE are more suitable for sample pre-treatment through MeOH PPT. The best detection results for HDHA were obtained after ACN PPT. Furthermore, EpOME, EpETE, EpDPA, and EET are suitable for the MTBE LLE protocol, while medium-chain HETEs are suitable for EA LLE.

#### 3.2.2. Extraction Recovery

**Figure 4:**
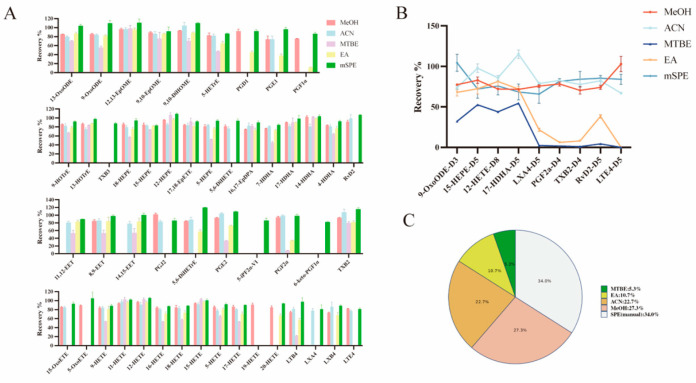
Summary of extraction recoveries obtained for standards spiked (25 ng/mL) in serum samples for each sample preparation protocol. (**A**) Comparison of extraction recoveries using different extraction methods. (**B**) Comparison of extraction recoveries of nine ISs using different extraction methods. (**C**) The extraction recovery rate of different methods ranges from 80% to 120%.

Extraction efficiency was evaluated by comparing protocol recovery rates for fifty reference metabolites and nine internal standards spiked into serum. PPT serves as an effective approach for simultaneous protein removal and lipid extraction from biological specimens. Previous studies [23,28,29] demonstrate that organic solvents including ACN and MeOH efficiently isolate free oxylipins from biological matrices. However, it is important to note that there are significant differences in the efficiency of these solvents for different oxylipin categories. For the HETrE, HEPE, ω-terminal HETE, DiHETE, DiHOME, DiHETrE, and HOTrE, which are advantage oxylipins extracting from MeOH, a high recovery rate was achieved (Figure 4A).

However, the EA and MTBE protocol extraction showed selective extraction that resulted in a poor recovery of LXs, PGs, TXs, Rvs, and LTs (Figure 4A). Similar issues have also existed in previous studies [30]. However, in our study, we can find that for the advantageous compounds in EA and MTBE schemes, medium-chain HETE and epoxy-oxylipins have satisfactory extraction recovery rates. Furthermore, in our research, the mSPE protocol extraction offered high recovery, such as for the MeOH and ACN protocols, which showed recoveries between 80.82% and 119.88% (Figure 4A). Meanwhile, the extraction recoveries of nine ISs proved the above results (Figure 4B). To intuitively compare the differences in the recoveries of the different extraction protocols, the proportion of oxylipins with recoveries between 80% and 120% in different extraction protocols are shown in Figure 4C. The results showed that mSPE protocols account for the largest proportion, suggesting that compared with the other four protocols, the mSPE protocols show the highest accuracy.

#### 3.2.3. Matrix Effects

The signal suppression or signal enhancement effects (both referred to as SSE) are due to the presence of co-eluting matrix components in the LC–MS/MS interface as described above. Although no clear guideline regarding the acceptable range of MEs has been provided for metabolite analysis to date, it has been suggested that they should be classified as soft (response of matrix-matched standard is ±20% compared with the solvent standard, i.e., SSE values range from 80 to 100% in the case of soft suppression and from 100 to 120% in the case of soft enhancement), moderate (from ±50 to ±20% relative to the solvent-based standard, i.e., SSE between 50 and 80% in the case of medium suppression and 120–150% in the case of medium enhancement) and strong (outside ±50% relative to the solvent-based standard, i.e., SSE < 50% in the case of strong suppression and >150% in the case of strong enhancement) [31]. While the MEs were soft for 72% of all investigated oxylipins for these five protocols, the number of oxylipins exhibiting medium or strong suppression or enhancement was significantly larger in the MeOH, ACN, MTBE, and EA protocols compared with the mSPE protocol (Table 1).

### 3.3. Optimization of the Preferred Extraction Protocol

#### 3.3.1. Comparison Between Manual and aSPE

Analysis of large batches of samples containing small amounts of material is challenging. In this study, we examined two SPE methods, viz. mSPE and aSPE. For consistency, the same type of SPE column was used for all methods.

**Figure 5:**
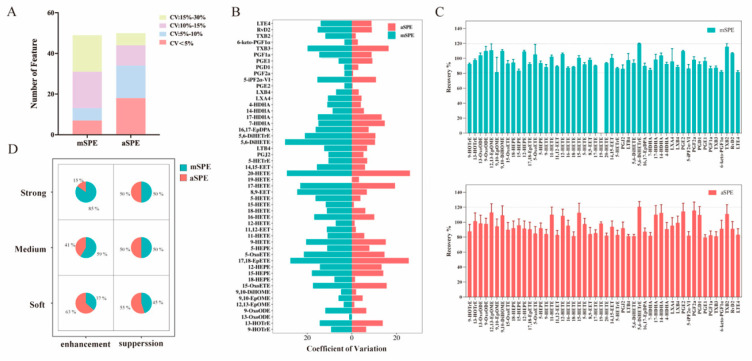
Comparison of mSPE and aSPE. (**A**) Histograms of detected features in each protocol are used to assess the extraction efficiency. (**B**) The coefficient of variation (CV%) of peak intensity for 50 compounds detected by both manual and aSPE. (**C**) Extraction recoveries with mSPE and aSPE. (**D**) Matrix pie chart of analytes attributed to various MEs.

As shown in Figure 5A, the difference between the number of oxylipins obtained using these two methods is small. However, the number of CVs < 10% in aSPE is greater than that in mSPE. The results showed that the aSPE method had better reproducibility. Furthermore, a comparison of the CV% values of 50 types of oxylipins that show peaks for both methods indicates that compared with mSPE, the aSPE method significantly improved the CV values of TXB2, 5,6-DiHETrE, 15-HETE, 5-HETE, 5,6-DiHETE, and 8,9-EET. This may be attributed to the lower exposure of the eluting solvent to the air, less adsorption of impurities by the solvent, and more thorough elution (Figure 5B).

In addition, with regard to the extraction recovery rate (Figure 5C), no significant differences were observed between the two methods. However, a comparison of the MEs (Figure 5D) of the two methods shows that aSPE results for more compounds are within the range of mild enhancement or inhibition (80–120%), indicating that the aSPE method is less affected by the MEs.

Our results demonstrate that the use of aSPE leads to better repeatability in the detection of oxylipins, although the improvement in recovery is not particularly noticeable due to the selection of elution solvent types. Of course, this conclusion does not hinder the use of aSPE platforms, because this method greatly reduces the processing time and is more suitable for large-scale metabolomics research.

#### 3.3.2. Optimization of SPE Elution Solvents and Reconstitution Solvents

After the comparison of different aspects of the extraction protocols, a final extraction protocol with 10% MeOH as the washing solvent and elution with MeOH, and MeOH as the reconstitution solvent was suggested. However, some issues remained unsolved such as the low extraction recovery for the eicosanes classes of PGs and LTs. To resolve these issues and increase the extraction rate of oxylipins, based on the above insights and results we designed another series of experiments to further fine-tune the best-performing extraction protocol. Here, the first step of using 10% MeOH as the washing solvent was modified by replacing MeOH with pure water. Further fine-tuning was performed to optimize the elution ability of PGs type oxylipins by adding 0.02% FA to the eluting solvent MeOH. It is worth noting that as the polarity of the eluent increases, the extraction efficiency also increases (Figure S3), which may be due to the increased amount of oxylipins present in the serum in a protonated form. Therefore, the acidification of the extraction solvent can lead to an enhancement of extraction ability.

**Figure 6:**
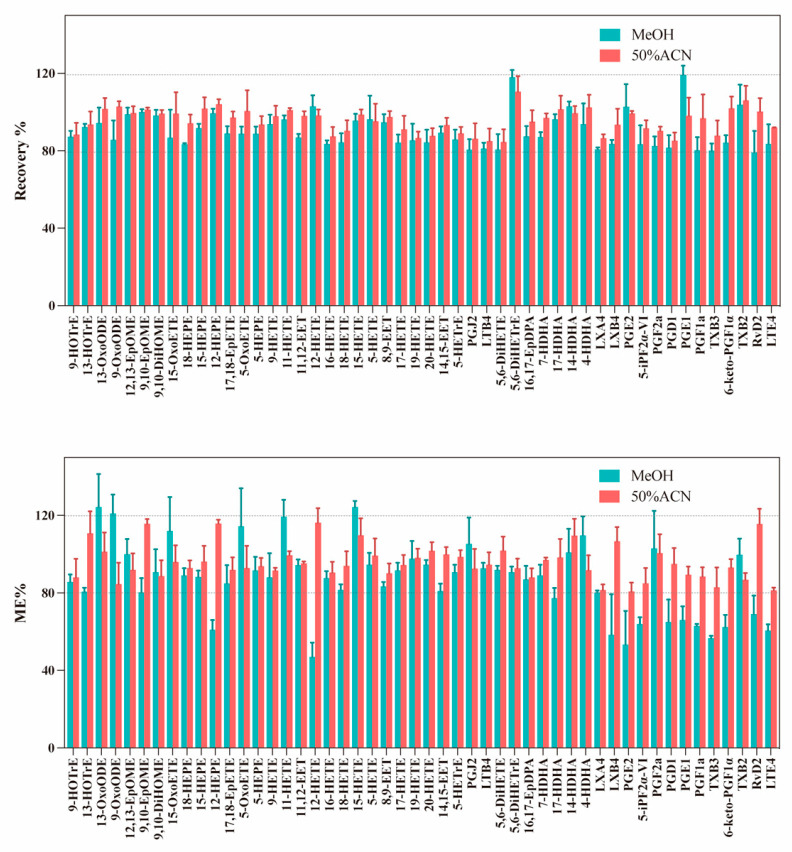
Comparison of the extraction recoveries obtained for 50 standards and MEs obtained for standards spiked (25 ng/mL) in serum samples with sample reconstituted by MeOH or 50% ACN (*n* = 3 for each reconstitution method).

Quantitative reconstitution is crucial for a robust workflow. Therefore, we investigated the effects of different complex solutions on the extraction recovery rate and ME, including MeOH and 50% ACN. As shown in Figure 6, compared to the MeOH protocol, all eicosanoids show close to 100% extraction recoveries in the 50% ACN reconstituting protocol. The same extraction recovery (close to 100%) is also observed for other oxylipins. In addition, the results are quite satisfying and the optimized protocol using 50% ACN does not exhibit significantly stronger MEs than MeOH. To conclude, the optimized protocol with 50% ACN can achieve the best results for all oxylipins classes including the eicosanoids. Furthermore, compared with pure MeOH, the use of 50% ACN as the reconstitution solvent not only improves the extraction recovery of oxylipins but also achieves better injection precision (with lower CV values) (Figure S4). This is likely attributable to its lower volatility and enhanced sample stability in the autosampler.

#### 3.3.3. Accuracy, Precision, Linearity, and Sensitivity

Apart from investigating the extraction recovery rate and matrix effect of the final aSPE–LC–MS/MS analytical method, this study further investigated the linearity, accuracy, precision, and sensitivity of the method to confirm its suitability for clinical sample testing. All analytes demonstrated method accuracy of 80.16–116.92% and precision ≤ 11.35% (Table S4). Calibration curves exhibited excellent linearity (r^2^ > 0.99, Table S5), with method sensitivity showing detection limits of 0.003–0.2 ng/mL (LOD) and quantification ranges of 0.009–0.56 ng/mL (LOQ, Table S5). The sensitivities of the present method of detecting oxylipins are comparable to other studies [32]. These results confirm that the assay’s accuracy and precision are sufficient for analyzing all oxylipin analytes in serum samples.

### 3.4. Serum Oxylipins Metabolomics Analyses Reveal Biomarkers of CHD

#### 3.4.1. Oxylipins Metabolic Profiling of Serum from CHD and HC

Method validation for biomarker detection was performed using pooled quality control (QC) samples created from 10 μL aliquots of each serum sample. These QC samples underwent identical pretreatment procedures alongside the analytical samples. During sample analysis, QC samples were analyzed after every 10 experimental samples to monitor system performance. The stability of the analytical system and method reproducibility were evaluated by calculating the relative standard deviation (RSD%) of internal standard peak areas in QC samples (Table S6), which ranged from 2.52% to 13.35%. These results revealed that this approach confirmed a high reproducibility and stability.

We performed oxylipin metabolic profiling in healthy and CHD participants. Classification of the serum samples was observed in the PLS-DA score-scatter plots derived from both the CHD and healthy groups, showing oxylipin metabolic alterations after CHD (Figure S5A). All R2Y and Q2 values from the PLS-DA models were greater than 0.5. The low values of intercepts R2 and Q2 showed that these models were not overfitted (Figure S5B).

#### 3.4.2. Differential Oxylipin Metabolites Between CHD and HC

**Figure 7:**
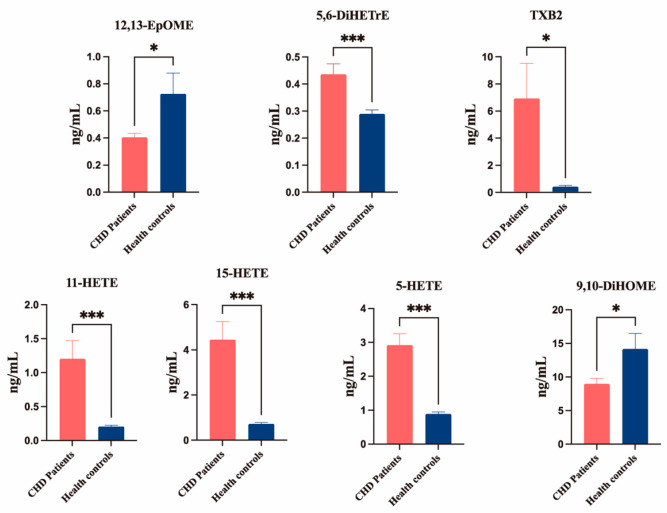
Serum oxylipins concentrations in the CHD and healthy control groups, as measured by UPLC–MS/MS. Data are means ± SEM. Results were compared using Student’s *t*-tests. *n* = 30–31 per group. * *p* < 0.05 and *** *p* < 0.001 vs. CHD group. *p*-value corrected by Bonferroni.

The selection of differential metabolites was based on stringent criteria: Bonferroni-corrected *p* < 0.05 and fold change thresholds (FC < 0.667 or >1.5). Analysis revealed five elevated oxylipin metabolites in CHD patients versus HCs: 11-HETE, 15-HETE, 5-HETE, TXB2, and 5,6-DiHETrE. Conversely, two metabolites showed significant reductions (12,13-EpOME and 9,10-DiHOME) in the CHD group (Figure 7).

#### 3.4.3. Potential Diagnostic Biomarker Selection and Integrated Biomarker Analysis

**Figure 8:**
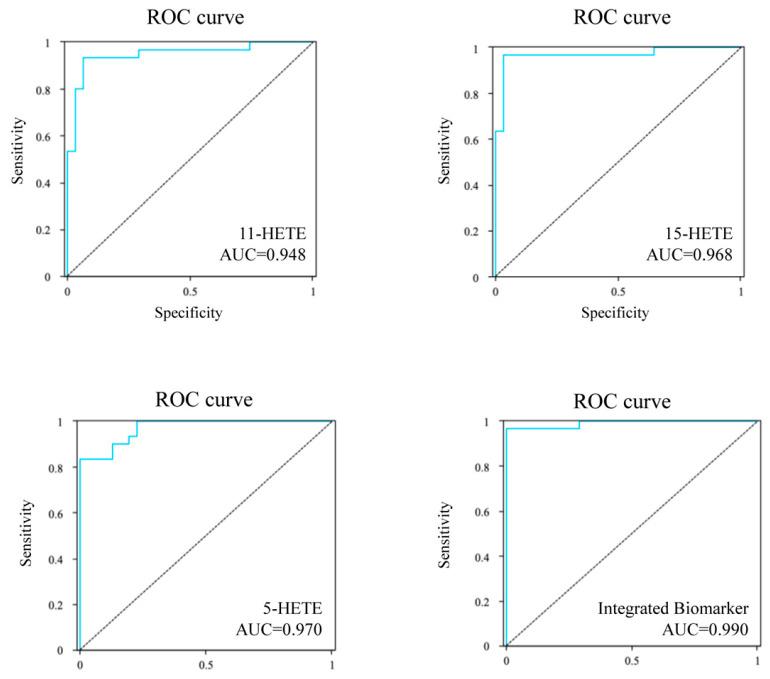
ROC curves of the 11-HETE, 15-HETE, 5-HETE, and the integrated biomarker for predicting the CHD.

To identify effective potential diagnostic biomarkers in serum metabolites of CHD, we conducted ROC curve analysis on seven differential metabolites. The findings are presented in Table 2, revealing five risk-elevating and two risk-reducing metabolites associated with CHD, aligning with the differential metabolite analysis results. Based on these findings, we selected the 11-HETE, 15-HETE, and 5-HETE as the top three biomarkers. The AUC values were 0.948 (95% CI, 0.890~1.007), 0.968 (95% CI, 0.920~1.015), and 0.970 (95% CI, 0.936~1.004), respectively (Figure 8). In this study, we further developed a comprehensive biomarker model by selecting these top three biomarkers with high AUC values (AUC > 0.99). As shown in Figure 8, our research indicates that 11-HETE, 15-HETE, and 5-HETE are comprehensive biomarkers for the diagnosis of CHD, with an AUC of 0.990, and have excellent sensitivity and specificity, with high clinical value, which can help patients with CHD receive early treatment.

## 4. Discussion

Oxylipins play a crucial role in various physiological functions. Different subtypes of oxylipins exhibit distinct chemical and biological properties [33,34,35,36,37,38,39,40,41,42]. However, currently, there is no optimal analysis protocol available for different oxylipin subtypes, and there is also a lack of high-throughput quantitative methods to measure oxylipins in the entire pathway. Therefore, to identify an optimal approach for oxylipin extraction, we first compared the efficiency of three techniques: PPT, LLE, and SPE.

Our results indicate that different subtypes of oxylipins have different advantages in pre-treatment strategies. In general, oxylipins have varying degrees of polarity, with epoxy-oxylipins being the least polar, followed by hydroxyl- and dihydroxy-oxylipins, which is positively related to the polarity of solvents used in different extraction methods. Among the solvents used for extraction, MeOH has the strongest hydrophilicity, followed by ACN, EA, and MTBE. Our research indicates that the differences in the primary extraction methods for different oxylipin subtypes are primarily determined by polarity. It is worth noting that medium-chain HETEs exhibit some hyperconjugation and spatial effects that reduce the hydrophilicity of hydroxyl groups. As a result, it yields better results when extracted using less polar EA, with acceptable ISs and standard recovery rates. On the other hand, this phenomenon is not observed in ω-terminal HETE, where the extraction efficiency is higher when using MeOH, which is a more polar solvent. Additionally, our results demonstrate that most types of eicosane compounds are efficiently extracted from serum samples using mSPE. This may be due to the low concentration of these oxylipins in serum samples. Not surprisingly, these data are consistent with the peak areas indicated in the ISs, suggesting that each type of oxylipin has its own extraction method that is optimally suitable for elution, as indicated by the results of our study. In conclusion, all extraction protocols share a large fraction overlap of extracted oxylipins, yet minor differences among the protocols exist. Moreover, each type of oxylipin has its own advantageous extraction protocols, providing a means for the future development of biomarkers for oxylipins. For example, Lu et al. showed that 9,10-DiHOME, 12,13-DiHOME, and 14,15-DiHETrE are correlated with the levels of tumor marker a-fetoprotei, which can be used as predictive indicators for hepatitis B virus-related liver disease [43]. As shown in Figure 3B, 9,10-DiHOME, 12,13-DiHOME, and 14,15-DiHETrE have the largest efficiency through the PPT method. Thus, in clinical testing of these three oxylipins, complex SPE methods are not required, and PPT can be directly used to obtain highly sensitive and efficient serum detection results. Given its broad coverage of oxylipins, the mSPE method was selected for further in-depth study and optimization.

A comparative study of mSPE and aSPE was conducted, which included an evaluation of three SPE and two re-dissolution solvents for their impact on target oxylipin determination. Based on the evaluation of the results obtained for internal standards using different testing protocols, such as the extraction efficiency, extraction recovery rate, and matrix effect, we recommend the following optimized extraction protocol. First, 50 μL serum should be extracted using an aSPE instrument, washing with 1.5 mL water, and eluting with 1.5 mL 0.02% FA in MeOH. Afterwards, re-dissolve in 50 μL of 50% ACN. This protocol provides the lowest sample consumption (50 μL serum), satisfying sensitivity (lower limit of quantitation at 0.01 to 0.5 ng/mL), high throughput (10 min for data acquisition), extraction efficiency (83.65–109.86%), low matrix effect (81.37–109.86%), satisfying accuracy (within 80.16–116.92%), and precision. Additionally, linear calibration curves of all analytes with r^2^ > 0.99 were obtained. Compared with previous studies [44], this method has successfully constructed a detection scheme for the entire pathway produced by different metabolic enzymes of oxylipins, which is of great significance for the discovery of biomarkers and therapeutic targets.

In addition, Mainka et al. showed that the targeted metabolomic analysis of total oxylipins exhibited substantial variability. Compared with it, this study excluded the variability caused by the personnel factors of the complex scheme, and the constructed automatic solid-phase extraction scheme itself had high repeatability (CV < 15% for 92% of all oxylipins) [45]. In addition, other research results have shown that ion inhibition has a significant impact on the detection of oxylipins by LC–MS [30]. However, this method showed a lower ion suppression effect, which may be attributed to smaller serum sample volume (50 μL) for short pre-treatment time and the aSPE–LC–MS/MS analytical platform providing a dark environment to prevent changes in the oxylipins. By reducing the introduction of foreign impurities, the efficient extraction of oxylipins can be achieved. Certainly, with the rapid technological advancements, the number of oxylipins that we are capable of detecting has been steadily increasing. However, the intricate diversity of oxylipins isomers poses significant challenges to current detection methodologies. For instance, Mainka et al. achieved the identification of 133 oxylipins, yet their method exhibited suboptimal reproducibility [45]. Wang et al.’s approach, though capable of detecting 183 oxylipins, displayed limited chromatographic resolution in separating HETE isomers [46]. It is noteworthy that the majority of methods capable of detecting over 100 oxylipins rely on the utilization of 100 μL to 200 μL of serum samples, which is undesirable for valuable clinical specimens due to the extensive sample requirements [20]. Despite this limitation, our study has focused on the detection of 50 oxylipins, which encompass all crucial metabolic pathways, providing a comprehensive insight into the physiological functions of oxylipins within the human body. This approach ensures a balanced assessment of the significance of these lipids while minimizing the impact on clinical sample availability. Collectively, this method greatly reduces the consumption of serum sample size [12,30], as well as labor costs, making it more suitable for large-scale metabolomics research and providing a more suitable solution for precious clinical sample detection.

CHD is a complex metabolic disorder caused by coronary atherosclerosis, which can lead to myocardial ischemia and even heart failure and has a high clinical mortality [47]. Oxylipins regulate inflammation, immunity, fibrosis, vasculature, reactive oxygen species accumulation, and mitochondrial function [2,48,49,50]. Additionally, cardiovascular pathologies such as CHD have been linked to aberrant oxylipin mediator signaling [51]. Therefore, the investigation of the changes in oxylipin metabolites in CHD is highly significant.

The selected protocol has been successfully applied to the targeted oxylipins metabolomics study of patients with CHD and provides an important means for the large-scale clinical studies of oxylipins. Dihydroxyicosatrienoic acid (DiHETrE) shows a positive association with cardiovascular event risk in peripheral arterial disease patients and demonstrates a strong predictive capacity [33,36]. Hydroxyeicosatetraenoic acid (HETE) has a chemotactic effect, which can alter vascular tension and induce the production of vascular endothelial growth factors, contributing to the pathogenesis of CHD [37,38,39,40]. Metabolomics studies have shown that LA is metabolized to epicyclooctadecanoic acids (EpOMEs) through CYP cyclooxygenase, and further metabolized to dihydroxyoctadecanoic acids (DiHOMEs) through soluble epoxide hydrolase (sEH). The inhibition of sEH can activate the PI3K signaling pathway and K^+^ channel while increasing the level of EpOMEs in the serum of acute myocardial infarction (AMI) to exert cardioprotective effects [34,41,43]. TXA2 plays a vital role in thrombosis and is responsible for the occlusion of blood vessels in CHD. Therefore, the pharmacological inhibition of TXA2 is an important target for CHD and related ischemic heart diseases [52]. It is reported that oxylipins metabolism has potential value to treat CHD, which provides a new perspective for the diagnosis and treatment of CHD. Taken together, these results suggest that the developed workflow was useful for differentiating between healthy and CHD individuals.

Compared with traditional heart detection techniques, biomarkers have advantages such as their early appearance, high specificity, and long diagnostic window. With the continuous addition of more biomarkers, we can achieve multi-biomarker analysis to improve the diagnostic efficiency of CHD. However, there are still some shortcomings in this study. To achieve the clinical value of biomarkers for CHD, we still need a larger sample size. In addition, the changes in biomarkers during the process of CHD are also worth further exploration.

## 5. Conclusions

In summary, for the first time, this study comprehensively analyzed different subtypes of oxylipins, including determining the optimal analytical protocol for each kind of oxylipin subtypes and establishing a high-throughput aSPE–LC–MS/MS analytical platform for a broad panel of complex oxylipin subtypes. Furthermore, the selectivity of the presented methods towards different subtypes of oxylipins were found and revealed based on the physicochemical principles of the chemical. Eventually, the platform was applied to detect serum samples of CHD patients, which discovered and provided a highly sensitive and specific set of combined serum biomarkers (5-HETE, 11-HETE, and 15-HETE) to diagnose CHD. The study provides the best protocol for the clinically targeted detection of oxylipins and provides an important means for studying the biomarkers of diseases.

## Figures and Tables

**Table 1:** Number of analytes attributed to various classes of ME.

	MeOH	ACN	MTBE	EA	mSPE
Soft suppression	31	14	5	2	12
Medium suppression	2	8	0	3	0
Strong suppression	6	11	1	1	0
Soft enhancement	4	9	14	5	24
Medium enhancement	0	0	6	20	7
Strong enhancement	0	0	7	8	6

**Table 2:** Diagnostic performance of 6 differential oxylipins metabolites and an integrated biomarker in predicting CHD by ROC analysis.

	AUC	Optimal Cut-Point Value	Sensitivity	Specificity	Cut-Off	SD	*p*	95%CI
11-HETE	0.948	0.869	0.933	0.935	0.282	0.030	<0.001 **	0.890~1.007
15-HETE	0.968	0.934	0.967	0.968	1.096	0.024	<0.001 **	0.920~1.015
5-HETE	0.970	0.833	0.833	1.000	1.554	0.017	<0.001 **	0.936~1.004
5.6-DiHETrE	0.734	0.440	0.633	0.806	0.341	0.066	0.002 **	0.606~0.863
TXB2	0.838	0.604	0.733	0.871	0.430	0.051	<0.001 **	0.737~0.938
12,13-EpOME	0.651	0.253	0.419	0.833	0.520	0.070	0.043 *	0.513~0.788
9,10-DiHOME	0.706	0.470	0.903	0.567	7.444	0.068	0.006 **	0.573~0.840
Integrated Biomarker	0.990	0.967	1.000	0.967	0.121	0.010	<0.001 **	0.970~1.010

* *p* < 0.05. ** *p* < 0.01.

## Data Availability

The data used and/or analyzed to support the findings of this study are available in this paper or the Supplementary Information. Any other raw data that support the findings of this study are available from the corresponding author upon reasonable request.

## Supplementary Materials

The following supporting information can be downloaded at https://www.mdpi.com/article/10.3390/metabo16010004/s1, Figure S1: Six sample preparation protocols were evaluated; Figure S2: The oxylipins extraction protocol optimized workflow; Figure S3: Peak area of oxylipins under different SPE washing and elution solvent conditions; Figure S4: Coefficient of variation (CV%) of samples reconstituted with different solvents; Figure S5: Partial least squares discriminant analysis (PLS-DA) plots (A) and permutation test (*n* = 200) (B) of the PLS-DA model were generated based on serum metabolic profiles detected in negative ion modes between CHD patients and healthy control groups; Table S1: Details regarding the standards used in this study; Table S2: Precursor and product ions (*m*/*z*) for the targeted oxylipins with corresponding MS detection conditions; Table S3: Baseline study participant characteristics; Table S4: Precision and accuracy data for oxylipins in SQC samples; Table S5: Calibration curves, LODs, and LOQs for analysis of oxylipin standards; Table S6: Repeatability results for internal standards in oxylipins metabolism in serum from CHD patients.

## Abbreviations

The following abbreviations are used in this manuscript:

AA	Arachidonic acid
ACN	Acetonitrile
ALA	A-linoleic acid
AMI	Acute myocardial infarction
aSPE	Automated SPE
CHD	Coronary heart disease
COX	Cyclooxygenase
CV	Coefficient of variation
CYP450	Cytochrome P450
DHA	Docosahexaenoic acid
DiHETrE	Dihydroxyicosatrienoic acid
DiHOMEs	Dihydroxyoctadecanoic acids
EA	Ethyl acetate
EPA	Eicosapentaenoic acid
EpOMEs	Epicyclooctadecanoic acids
ESI	Electrospray ionization
FA	Formic acid
HC	Health control
HETE	Hydroxyeicosatetraenoic acid
HLB	Hydrophilic–lipophilic balance
IPA	Isopropanol
IS	Internal standard
LA	Linoleic acid
LC–MS	Liquid chromatography–mass spectrometry
LLE	Liquid–liquid extraction
LOX	Lipoxygenase
LVEF	Left ventricular ejection function
ME	Matrix effect
MS/MS	Random mass spectrometry
mSPE	Manual SPE
MTBE	Methyl tert-butyl ether
PCA	Principal component analysis
PLS-DA	Partial least squares discriminant analysis
PPT	Protein precipitation
PUFA	Polyunsaturated fatty acid
sEH	Soluble epoxide hydrolase
SPE	Solid-phase extraction
SQC	Standard quality control
UPLC	Ultra-performance liquid chromatography
