# Supplementary material for: Comprehensive Analysis of Different Subtypes of Oxylipins to Determine a LC–MS/MS Approach in Clinical Research
Source: Metabolites. 2025 Dec 22;16(1):4. doi: 10.3390/metabo16010004 (PMC12843908; doi:10.3390/metabo16010004)
Supplement: Supplementary file 1 [file metabolites-16-00004-s001.zip › metabolites-4057791-supplementary.pdf]

Supplementary Methods and Data for

Comprehensive analysis of different subtypes of oxylipins to determine a LC−MS/MS approach in clinical research.

1. Optimization of aSPE extraction efficiency

1.1. Optimization of aSPE eluent solution

To optimize the extraction efficiency of aSPE, the serum was divided into nine aliquots for the examination of three extraction solvents. In the first scheme, 10% MeOH was the washing solvent and MeOH was the elution solvent. The second solution used pure water as the washing solvent and eluted with MeOH. In the third scheme, pure water was used as the washing solvent and MeOH containing 0.02% FA was used for elution. Each protocol was evaluated with three replicates. Based on the chromatographic peak intensity **(Fig. S3)**, the result showed that the extraction efficiency of oxylipins was optimal under the conditions of washing with 1.5 mL pure water and eluting with 1.5 mL 0.02% formic acid in MeOH (Section 2.3.4).

1.2. Automatic SPE extraction method

The serum was divided into six aliquots for two different reconstitution solvents with three replicates for each using the third aSPE protocol described in supplementary section 1.1. After drying the extraction solvent, the dried oxylipin extracts were then reconstituted in MeOH (50 μL), or H_2O/ACN (1:1; v/v，50μL). In the last step, the reconstituted samples were vortexed (2 min), centrifuged (13225 g, 5 min, 4 °C) and transferred to autosampler vials for storing at -20 °C prior to analysis. The oxylipins extraction protocol optimized workflow is presented in **Fig. S2**.

2. Patient Enrollment

Inclusion criteria for patients with coronary heart disease: (1) The patient meets the diagnostic criteria for stable angina pectoris, (2) NYHA's cardiac function

classification is I ~ II, (3) 40 years old ≤ age ≤ 75 years old; (4) The subject is informed and voluntarily signs an informed consent form. Exclusion criteria: (1) Renal dysfunction, male serum creatinine>2.5 mg/dL (>220umol/L), female serum creatinine>2.0 mg/dL (>175umol/L); (2) Suffering from obvious liver diseases or having ALT and AST levels three times higher than the normal upper limit; (3) Patients with poor blood pressure control (systolic blood pressure>160 mmHg or diastolic blood pressure>100 mmHg after taking antihypertensive drugs) need to sit for at least 5 minutes before measuring blood pressure); (4) Patients with severe diabetes metabolic syndrome; (5) Patients with severe chronic heart failure, severe arrhythmia, or a history of installing a pacemaker or myocardial infarction; (6) The patient has active gastrointestinal ulcers and other hemorrhagic diseases; (7) Patients with malignant tumors, autoimmune diseases, or hematological disorders; (8) Patients with mental illness; (9) Individuals who are allergic to known components of the study drug; (10) Pregnant or expectant women, lactating women; (11) Those who have participated or are currently participating in other clinical trials within the past three months. Serum oxylipins levels were measured in all participants as detailed above.

3. Ethics approval and consent to participate

All animal studies were performed following the guidelines of the Beijing Municipal Ethics Committee for the care and use of laboratory animals and were approved by the Animal Care & Welfare Committee, Chinese Academy of Medical Sciences (IRM-DWLL-2021114) (Beijing, China) . The Ethics Committee of the Medical Experimental Center of the Chinese Academy of Chinese Medical Sciences approved the collection of samples, and written informed consent was obtained from each subject. All patient studies were approved by the ethics committee of the Medical Experimental Center of the Chinese Academy of Chinese Medical Sciences (WJEC-

KT-2021-032-P003) and was conducted under the restriction of the ethical guidelines of the 1975 Declaration of Helsinki. Written informed consent was obtained from all participants included in the study.

Table captions

Table S1. Details regarding the standards used in this study.

Table S2. Precursor and Product Ions (m/z) for the targeted oxylipins with corresponding MS detection conditions.

Table S3. Baseline study participant characteristics.

Table S4. Precision and accuracy data for oxylipins in SQC samples.

Table S5. Calibration curves, LODs and LOQs for analysis of oxylipin standards.

Table S6. Repeatability results for internal standards in Oxylipins metabolism in serum from CHD patients.

Figure captions

Figure S1. Six sample preparation protocols were evaluated. (A) Protocol 1: Methanol protein precipitation. (B) Protocol 2: Acetonitrile protein precipitation. (C) Protocol 3: Ethyl acetate extraction protocol. (D) Protocol 4: Methyl tertbutyl ether extraction protocol. (D) Protocol 5: Manual Oasis HLB Cartridge-SPE extraction protocol. (E) Protocol 6: Automatic Oasis HLB Cartridge-SPE extraction protocol.

Figure S2. The oxylipins extraction protocol optimized workflow.

Figure S3. Peak area of oxylipins under different SPE washing and elution solvent conditions.

Figure S4. Coefficient of Variation (CV%) of Samples Reconstituted with Different Solvents.

Figure S5. Partial least squares discriminant analysis (PLS-DA) plots. (A) and permutation test (n=200) (B) of the PLS-DA model were generated based on serum metabolic profiles detected in negative ion modes between CHD patients and healthy control groups.

Table S1. Details regarding the standards used in this study.

CAS No.	Analytes	Abbreviations	Source
58962-34-8	6-keto Prostaglandin F1α	6-keto-PGF1α	Cayman
71953-80-5	Thromboxane B3	TXB3	Cayman
54397-85-2	Thromboxane B2	TXB2	Cayman
179094-11-2	(±)5-iPF2α-VI	5-iPF2α-VI	Cayman
745-62-0	Prostaglandin F1α	PGF1α	Cayman
57944-39-5	1a,1b-dihomo Prostaglandin F2α	PGF2α	Cayman
363-24-6	Prostaglandin E2	PGE2	Cayman
98049-69-5	Lipoxin B4	LXB4	Cayman
745-65-3	Prostaglandin E1	PGE1	Cayman
17968-82-0	Prostaglandin D1	PGD1	Cayman
810668-37-2	Resolvin D2	RvD2	Cayman
89663-86-5	Lipoxin A4	LXA4	Cayman
75715-89-8	Leukotriene E4	LTE4	Cayman
60203-57-8	Prostaglandin J2	PGJ2	Cayman
71160-24-2	Leukotriene B4	LTB4	Cayman
263399-34-4	(±)-Threo-9,10-dihydroxy-12(Z)-octadecenoic acid	9,10-DiHOME	Cayman
845673-97-4	5,6-Dihydroxy-8Z,11Z,14Z,17Z-eicosatetraenoic acid	5,6-DiHETE	Cayman
89886-42-0	9-Hydroxy-10,12,15-octadecatrienoic acid	9-HOTrE	Cayman

141110-17-0	18-hydroxy-5(Z),8(Z),11(Z),14(Z),16(E)-eicosapentaenoic acid	18-HEPE	Cayman
87984-82-5	13S-hydroxy-9Z,11E,15Z-octadecatrienoic acid	13-HOTrE	Cayman
115461-40-0	(19S)-Hydroxyeicosatetraenoic acid	19-HETE	Cayman
88852-33-9	15-hydroxy-5(Z),8(Z),11(Z),13(E),17(Z)-eicosapentaenoic acid	15-HEPE	Cayman
79551-86-3	20-Hydroxyeicosatetraenoic acid	20-HETE	Cayman
133268-58-3	18-hydroxy-5Z,8Z,11Z,14Z-eicosatetraenoic acid	18-HETE	Cayman
81187-21-5	12-Hydroxy-5,8,10,14,17-eicosapentaenoic acid	12-HEPE	Cayman
128914-47-6	17-hydroxy-5Z,8Z,11Z,14Z-eicosatetraenoic acid	17-HETE	Cayman
213382-49-1	5,6-dihydroxy-8Z,11Z,14Z-eicosatrienoic acid	5,6-DiHETrE	Cayman
128914-46-5	16-hydroxy-5Z,8Z,11Z,14Z-eicosatetraenoic acid	16-HETE	Cayman
83952-40-3	5-Hydroxyeicosapentaenoic acid	5-HEPE	Cayman
131339-23-6	17,18-Epoxy-5,8,11,14-eicosatetraenoic acid	17,18-EpETE	Cayman
54739-30-9	13-Oxo-9E,11E-octadecadienoic acid	13-OxoODE	Cayman

71030-36-9	15-hydroxy-5Z,8Z,11Z,13E-eicosatetraenoic acid	15-HETE	Cayman
90780-52-2	17-Hydroxydocosahexaenoic acid	17-HDHA	Cayman
81416-72-0	(5Z,8Z,11Z,13E)-15-oxoicosa-5,8,11,13-tetraenoic acid	15-OxoETE	Cayman
54232-59-6	9-Oxo-10(E),12(Z)-octadecadienoic acid	9-OxoODE	Cayman
119433-37-3	14(S)-Hydroxy Docosahexaenoic Acid	14-HDHA	Cayman
54886-50-9	(5Z,8Z,11S,12E,14Z)-11-hydroxyicosa-5,8,12,14-tetraenoic acid	11-HETE	Cayman
71030-37-0	12-hydroxy-5Z,8Z,10E,14Z-eicosatetraenoic acid	12-HETE	Cayman
90780-55-5	7-hydroxydocosa-4,8,10,13,16,19-hexaenoic acid	7-HDHA	Cayman
79495-85-5	9-hydroxy-5Z,7E,11Z,14Z-eicosatetraenoic acid	9-HETE	Cayman
73307-52-5	5-Hydroxyeicosatetraenoate	5-HETE	Cayman
6799-85-5	12,13-epoxy-9-octadecenoic acid	12,13-EpOME	Cayman
197508-62-6	14,15-epoxy-5Z,8Z,11Z-eicosatrienoic acid	14,15-EET	Cayman
155073-46-4	(±)16(17)-EpDPA	16,17-EpDPA	Cayman
65167-83-1	9,10-Epoxy-12-octadecenoic acid	9,10-EpOME	Cayman

90906-40-4	(+/-)-4-hydroxy-5E,7Z,10Z,13Z,16Z,19Z-docosahexaenoic acid	4-HDHA	Cayman
106154-18-1	5-oxo-6E,8Z,11Z,14Z-eicosatetraenoic acid	5-OxoETE	Cayman
123931-40-8	11,12-epoxyeicosatrienoic acid	11,12-EET	Cayman
184488-44-6	8,9-epoxy-5Z,11Z,14Z-eicosatrienoic acid	8,9-EET	Cayman
195061-94-0	5S-hydroxy-6E,8Z,11Z-eicosatrienoic acid	5-HETrE	Cayman
1346112-79-5	Thromboxane B2-d4	TXB2-D4	Cayman
34210-11-2	Prostaglandin F2alpha-d4	PGF2a-D4	Cayman
1881277-33-3	Resolvin D2-d5	RvD2-D5	Cayman
1622429-53-1	Lipoxin A4-d5	LXA4-D5	Cayman
1240398-14-4	Leukotriene E4-d5	LTE4-D5	Cayman
2750534-74-6	15(S)-hydroxy Eicosapentaenoic Acid-d5	15-HEPE-D5	Cayman
2738376-87-7	17(S)-Hydroxy docosahexaenoic acid-d5	17-HDHA-D5	Cayman
2714198-70-4	9-oxo-10E,12Z-10,12,13-d3-octadecadienoic acid	9-OxoODE-D3	Cayman

84807-90-9	12S-hydroxy-5Z,8Z,10E,14Z-eicosatetraenoic acid-d8	12-HETE-D8	Cayman

Table S2. Precursor and Product Ions (m/z) for the targeted oxylipins with corresponding MS detection conditions.

Compound	Precursor ion	Daughter Ion	RT	CV	CE
6-keto-PGF1α	369.1	207	0.81	25	16
TXB3	367.1	169	0.87	25	14
TXB2	369.1	169	1.01	25	14
5-iPF2α-VI	353.28	115.05	1.1	40	22
PGF1α	355.3	293.2	1.12	22	20
PGF2α	353.28	193.1	1.13	40	24
PGE2	351.3	333.2	1.32	40	12
LXB4	351.28	233.1	1.4	25	16
PGE1	353.28	317	1.28	40	16
PGD1	353.28	235.15	1.37	40	16
RvD2	375.2	175	1.36	25	20
LXA4	351.1	115	1.66	25	16
LTE4	438.1	333.1	2.11	25	16
PGJ2	333.3	189.1	2.22	40	16
LTB4	335.02	195.1	3.22	25	14
9,10-DiHOME	313.28	201.06	6.56	12	20
5,6-DiHETE	335.28	145.09	4.12	2	18
9-HOTrE	293.21	171.1	4.75	4	14
18-HEPE	317.21	215.2	4.85	4	16
13-HOTrE	293.28	195.22	4.87	28	16
19-HETE	319.3	231.1	5.08	20	14

15-HEPE	317.28	219.16	5.25	2	12
20-HETE	319.3	289.3	5.27	12	16
18-HETE	319.28	261.23	5.44	2	16
12-HEPE	317.28	179.1	5.57	10	14
17-HETE	319.28	247.24	5.57	16	12
5,6-DiHETrE	337.34	145.08	5.6	2	18
16-HETE	319.28	233.1	5.68	4	12
5-HEPE	317.28	115.01	6.04	22	20
17,18-EpETE	317.28	215.19	6.23	6	14
13-OxoODE	293.28	113.1	6.32	2	22
15-HETE	319.28	219.15	6.32	4	12
17-HDHA	343.28	245.1	6.42	2	12
15-OxoETE	317.2	113.1	6.56	12	16
9-OxoODE	293.28	185.08	6.7	4	20
14-HDHA	343.28	205.1	6.71	4	12
11-HETE	319.28	167.07	6.73	4	16
12-HETE	319.28	179.1	6.95	2	12
7-HDHA	343.28	141.1	7.14	4	12
9-HETE	319.1	123	7.22	20	14
5-HETE	319.28	301.29	7.57	2	8
12,13-EpOME	295.21	195.15	7.61	2	16
14,15-EET	319.34	219.16	7.68	22	4
16,17-EpDPA	343.21	233.21	7.82	30	12
9,10-EpOME	295.28	277.21	7.83	8	14
4-HDHA	343.28	101	7.85	4	14

5-OxoETE	317.28	203.17	8.13	2	20
11,12-EET	319.28	167.07	8.19	4	16
8,9-EET	319.28	155.2	8.39	20	12
5-HETrE	321.3	115	9.24	20	15
TXB2-D4#	373	173	1.01	25	14
PGF2a-D4#	357	197	1.13	40	24
RvD2-D5#	380	175	1.35	25	20
LXA4-D5#	356	115	1.64	25	16
LTE4-D5#	443	338	2.09	25	16
15-HEPE-D5#	322	219	5.2	2	12
17-HDHA-D5#	348	286.3	6.38	2	12
9-OxoODE-D3#	296	185.08	6.65	4	20
12-HETE-D8#	327	184	6.85	2	12

# represents internal standard

Table S3. Baseline study participant characteristics.

	CHD patients (n=31)	Healthy controls (n=30)	p value
**Demographic characteristics**			
Age, years	62.87±7.33	62.74±5.03	0.94
Sex, male	17(54.84)	23 (78.95）	0.09
Systolic blood pressure (mmHg)	133.36±14.64	138.11±21.74	0.36
Diastolic blood pressure (mmHg)	80.68±9.06	80.84±15.49	0.96
**Laboratory parameters**			
Total cholesterol (mmol/L)	4.27±0.90	4.47±0.68	0.43
Triglycerides	1.45±1.01	1.102±0.43	0.16
Creatinine	76.32±28.22	79.32±14.87	0.67
NT-proBNP	1.31±0.27	1.37±0.26	0.42
LVEF (%)	2.43±0.76	2.21±0.63	0.30

Data are means ± SEM and were compared using Student’s t-tests. Chi-squared tests were used to compare categorical variable.

Table S4. **Precision and accuracy data for oxylipins in SQC samples.**

Compounds	Spiked conc.	Peak area (Mean± SD)	Precision (CV%)	Accuracy (%)
6-keto-PGF1α	1ng/mL	3916.83±343.53	8.77	110.06±4.03
TXB3	1ng/mL	4716.66±224.86	4.77	98.88±1.76
TXB2	1ng/mL	10669.27±661.27	6.2	103.36±5.94
5-iPF2α-VI	1ng/mL	3792.25±430.31	11.35	84.75±3.34
PGF1α	1ng/mL	5391.09±54.31	1.01	98.14±5.77
PGF2α	1ng/mL	6222.11±49.06	0.79	93.46±7.74
PGE2	1ng/mL	12906.08±1038	8.04	108.4±1.93
LXB4	1ng/mL	1075.53±109.63	10.19	104.7±9.53
PGE1	1ng/mL	5614.17±394.66	7.03	99.6±5.78
PGD1	1ng/mL	3176.34±58.07	1.83	101.11±4.79
RvD2	1ng/mL	3030.23±32.22	1.06	97.08±3.85

LXA4	1ng/mL	5116.02±288.59	5.64	86.39±7.22
LTE4	1ng/mL	3887.33±146.95	3.78	113.05±3.87
PGJ2	1ng/mL	772.17±52.44	6.79	88.74±8.45
LTB4	1ng/mL	7980.17±158.29	1.98	90.05±8.52
9,10-DiHOME	1ng/mL	17844.67±21.33	0.12	84.9±1.93
5,6-DiHETE	1ng/mL	4707.24±338.29	7.19	84.77±2.89
9-HOTrE	1ng/mL	6437.81±207.49	3.22	95.42±6.36
18-HEPE	1ng/mL	2084.59±73.9	3.54	87.37±4.85
13-HOTrE	1ng/mL	3165.39±235.99	7.46	93.52±1.87
19-HETE	1ng/mL	1177.25±94.21	8	91.67±11.75
15-HEPE	1ng/mL	3114.68±79.92	2.57	100.68±1.65
20-HETE	1ng/mL	1662.77±48.86	2.94	108.1±4.9
18-HETE	1ng/mL	5186.48±3.5	0.07	106.16±5.6
12-HEPE	1ng/mL	4629.21±189.66	4.1	85.71±3.77
17-HETE	1ng/mL	7979.95±186.48	2.34	87.69±2.51

5,6-DIHETrE	1ng/mL	7827.52±33.5	0.43	87.15±3.42
16-HETE	1ng/mL	6233.62±16.64	0.27	85.64±3.03
5-HEPE	1ng/mL	3019.52±279.87	9.27	85.63±4.15
17,18-EpETE	1ng/mL	1904.99±10.13	0.53	102.53±17.18
13-oxoODE	1ng/mL	6612.3±132.6	2.01	85.34±5.6
15-HETE	1ng/mL	3194.02±232.24	7.27	87.8±7.5
17-HDHA	1ng/mL	890.98±60.07	6.74	87.67±1.81
15-oxoETE	1ng/mL	11191.11±434.23	3.88	90.29±3.01
9-oxoODE	1ng/mL	6416.74±114.16	1.78	91.49±5.04
14-HDHA	1ng/mL	2464.64±47.96	1.95	86.58±1.78
11-HETE	1ng/mL	18630.37±529.03	2.84	86.91±4.9
12-HETE	1ng/mL	13630.68±1277.84	9.37	109.07±0.54
7-HDHA	1ng/mL	4433.17±150.2	3.39	88.27±2.93
9-HETE	1ng/mL	1768.67±167.18	9.45	92.92±5.3
5-HETE	1ng/mL	13506.06±74.89	0.55	103.78±2.12

12,13-EpOME	1ng/mL	5196.05±31.03	0.6	87.17±4.74
14,15-EET	1ng/mL	1282.71±6.27	0.49	87.97±5.1
16,17-EpDPA	1ng/mL	3302.37±4.57	0.14	89.05±1.77
9,10-EpOME	1ng/mL	17614.34±612.84	3.48	97.11±6.45
4-HDHA	1ng/mL	7159.95±255.87	3.57	82.05±1.89
5-oxoETE	1ng/mL	3472.83±9.03	0.26	103.54±3.69
11,12-EET	1ng/mL	5454.95±129.4	2.37	87.76±4.89
8,9-EET	1ng/mL	2279.32±138.07	6.06	92.21±6.65
5-HETrE	1ng/mL	6264.02±373.35	5.96	82.44±1.01

Table S5. Calibration curves, LODs and LOQs for analysis of oxylipin standards.

Analyte	Calibration curves	r^2	LOD (ng/mL)	LOQ (ng/mL)	LLOQ (ng/mL)	Dynamic range(ng/mL)	IS
6-keto-PGF1α	y=1.302x+0.151	0.995	0.057	0.174	0.100	0.195-100	PGF2a-d4
PGJ2	y=0.148x+0.002	0.993	0.007	0.020	0.100	0.195-100	PGF2a-d4
TXB3	y=1.344x+0.073	0.995	0.027	0.081	0.025	0.049-100	TXB2-d4
5-iPF2α-VI	y=1.185x+0.058	0.992	0.024	0.073	0.070	0.098-100	PGF2a-d4
PGF1α	y=1.184x+0.094	0.991	0.039	0.119	0.050	0.098-100	PGF2a-d4
PGF2α	y=1.637x+0.053	0.992	0.016	0.049	0.072	0.098-100	PGF2a-d4
PGE2	y=27.827x+1.614	0.993	0.029	0.087	0.1	0.195-100	PGF2a-d4
LXB4	y=0.717x+0.219	0.995	0.151	0.458	0.100	0.195-100	LXA4-d5
PGE1	y=0.499x+0.012	0.998	0.012	0.036	0.060	0.098-100	PGF2a-d4
PGD1	y=0.843x+0.063	0.992	0.037	0.112	0.030	0.049-100	PGF2a-d4
TXB2	y=0.913x-0.021	0.998	-0.011	-0.035	0.080	0.098-100	TXB2-d4

LXA4	y=0.588x+0.008	0.994	0.007	0.020	0.010	0.024-100	LXA4-d5
LTE4	y=0.802x+0.145	0.993	0.089	0.271	0.050	0.098-100	LTE4-d5
RvD2	y=0.285x+0.041	0.993	0.071	0.216	0.120	0.195-100	RvD2-d5
LTB4	y=2.735x+0.176	0.991	0.032	0.097	0.015	0.024-100	LTE4-d5
9,10-DiHOME	y=4.297x+0.196	0.991	0.023	0.068	0.035	0.049-100	12-HETE-d8
5,6-DiHETE	y=0.663x+0.020	0.991	0.015	0.045	0.160	0.195-100	12-HETE-d9
9-HOTrE	y=0.773x+0.0451	0.992	0.029	0.088	0.012	0.024-100	12-HETE-d8
18-HEPE	y=0.454x+0.161	0.995	0.176	0.532	0.400	0.781-100	15-HEPE-d5
13-HOTrE	y=0.648x+0.037	0.994	0.028	0.086	0.025	0.049-100	12-HETE-d8
19-HETE	y=0.248x+0.093	0.996	0.186	0.563	0.500	0.781-100	12-HETE-d8
15-HEPE	y=0.927x+0.187	0.996	0.100	0.303	0.100	0.195-100	15-HEPE-d5
20-HETE	y=0.255x+0.016	0.998	0.031	0.094	0.150	0.195-100	12-HETE-d8
18-HETE	y=0.861x+0.063	0.992	0.036	0.110	0.030	0.049-100	12-HETE-d8
12-HEPE	y=0.912x+0.060	0.995	0.033	0.099	0.024	0.049-100	15-HEPE-d5
17-HETE	y=1.083x+0.125	0.994	0.057	0.173	0.060	0.098-100	12-HETE-d8

5,6-DiHETrE	y=1.24x+0.013	0.999	0.005	0.016	0.014	0.024-100	12-HETE-d9
16-HETE	y=1.156x+0.151	0.992	0.065	0.196	0.080	0.098-100	12-HETE-d8
5-HEPE	y=0.960x+0.079	0.996	0.041	0.123	0.050	0.098-100	15-HEPE-d5
17,18-EpETE	y=0.356x+0.075	0.996	0.104	0.316	0.500	0.781-100	15-HEPE-d5
13-OxoODE	y=13.998x+1.014	0.999	0.036	0.109	0.050	0.098-100	9-OxoODE-d3
15-HETE	y=1.131x+0.027	0.998	0.012	0.036	0.140	0.195-100	12-HETE-d8
17-HDHA	y=0.146x+0.028	0.994	0.095	0.288	0.100	0.195-100	17-HDHA-d5
15-OxoETE	y=24.690x+5.444	0.993	0.109	0.331	0.100	0.195-100	9-OxoODE-d3
9-OxoODE	y=12.124x+3.982	0.994	0.163	0.493	0.120	0.195-100	9-OxoODE-d3
14-HDHA	y=0.149x+0.002	0.996	0.007	0.020	0.030	0.049-100	17-HDHA-d6
11-HETE	y=6.889x+0.066	0.998	0.005	0.014	0.060	0.098-100	12-HETE-d8
12-HETE	y=1.294x+0.473	0.992	0.181	0.548	0.015	0.195-800	12-HETE-d8
7-HDHA	y=0.398x+0.010	0.994	0.012	0.038	0.030	0.049-100	17-HDHA-d7
9-HETE	y=0.375x+0.0342	0.994	0.045	0.137	0.080	0.098-100	12-HETE-d8
5-HETE	y=2.940x+0.018	0.998	0.003	0.009	0.360	0.391-100	12-HETE-d8

12,13-EpOME	y=16.019x+3.460	0.994	0.107	0.324	0.100	0.195-100	9-OxoODE-d3
14,15-EET	y=0.241x+0.037	0.993	0.076	0.230	0.180	0.195-100	12-HETE-d8
16,17-EpDPA	y=0.283x+0.017	0.995	0.030	0.090	0.064	0.098-100	17-HDHA-d8
9,10-EpOME	y=35.351x+5.790	0.999	0.081	0.246	0.200	0.391-100	9-OxoODE-d3
4-HDHA	y=0.624x+0.025	0.995	0.020	0.060	0.040	0.049-100	17-HDHA-d9
5-OxoETE	y=6.399x+1.967	0.993	0.152	0.461	0.100	0.195-100	9-OxoODE-d3
11,12-EET	y=0.779x+0.036	0.993	0.023	0.069	0.070	0.098-100	12-HETE-d8
8,9-EET	y=0.432+0.031	0.994	0.036	0.108	0.040	0.049-100	12-HETE-d8
5-HETrE	y=1.196x+0.024	0.994	0.010	0.030	0.020	0.024-100	12-HETE-d9

Note. CV (%) = SD×100 / Mean.

Table S6. Repeatability results for internal standards in Oxylipins metabolism in serum from CHD patients.

Ion mode	Compound	RSD(%)	RT(min)
ESI (-)	TXB2-D4#	5.28	1.01
ESI (-)	PGF2a-D4#	8.14	1.13
ESI (-)	RvD2-D5#	9.00	1.35
ESI (-)	LXA4-D5#	13.35	1.64
ESI (-)	LTE4-D5#	6.04	2.09
ESI (-)	15-HEPE-D5#	2.98	5.2
ESI (-)	17-HDHA-D5#	3.73	6.38
ESI (-)	9-OxoODE-D3#	2.52	6.65
ESI (-)	12-HETE-D8#	6.73	6.85

# represents internal standard

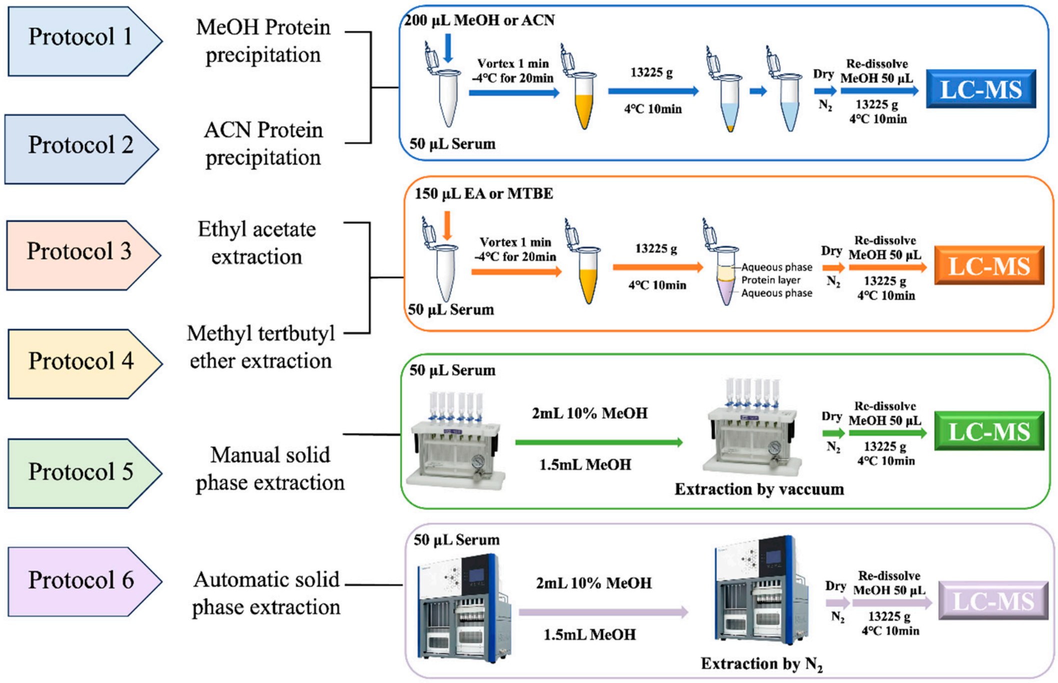

Figure S1. Six sample preparation protocols were evaluated. (A) Protocol 1: Methanol protein precipitation. (B) Protocol 2: Acetonitrile protein precipitation. (C) Protocol 3: Ethyl acetate extraction protocol. (D) Protocol 4: Methyl tertbutyl ether extraction protocol. (D) Protocol 5: Manual Oasis HLB Cartridge-SPE extraction protocol. (E) Protocol 6: Automatic Oasis HLB Cartridge-SPE extraction protocol.

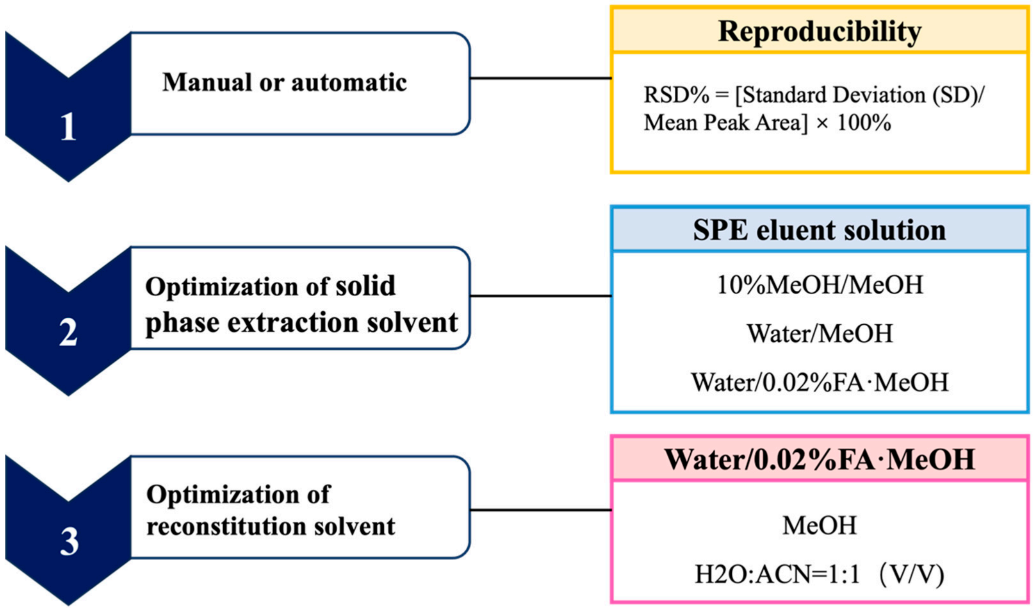

Figure S2. The oxylipins extraction protocol optimized workflow.

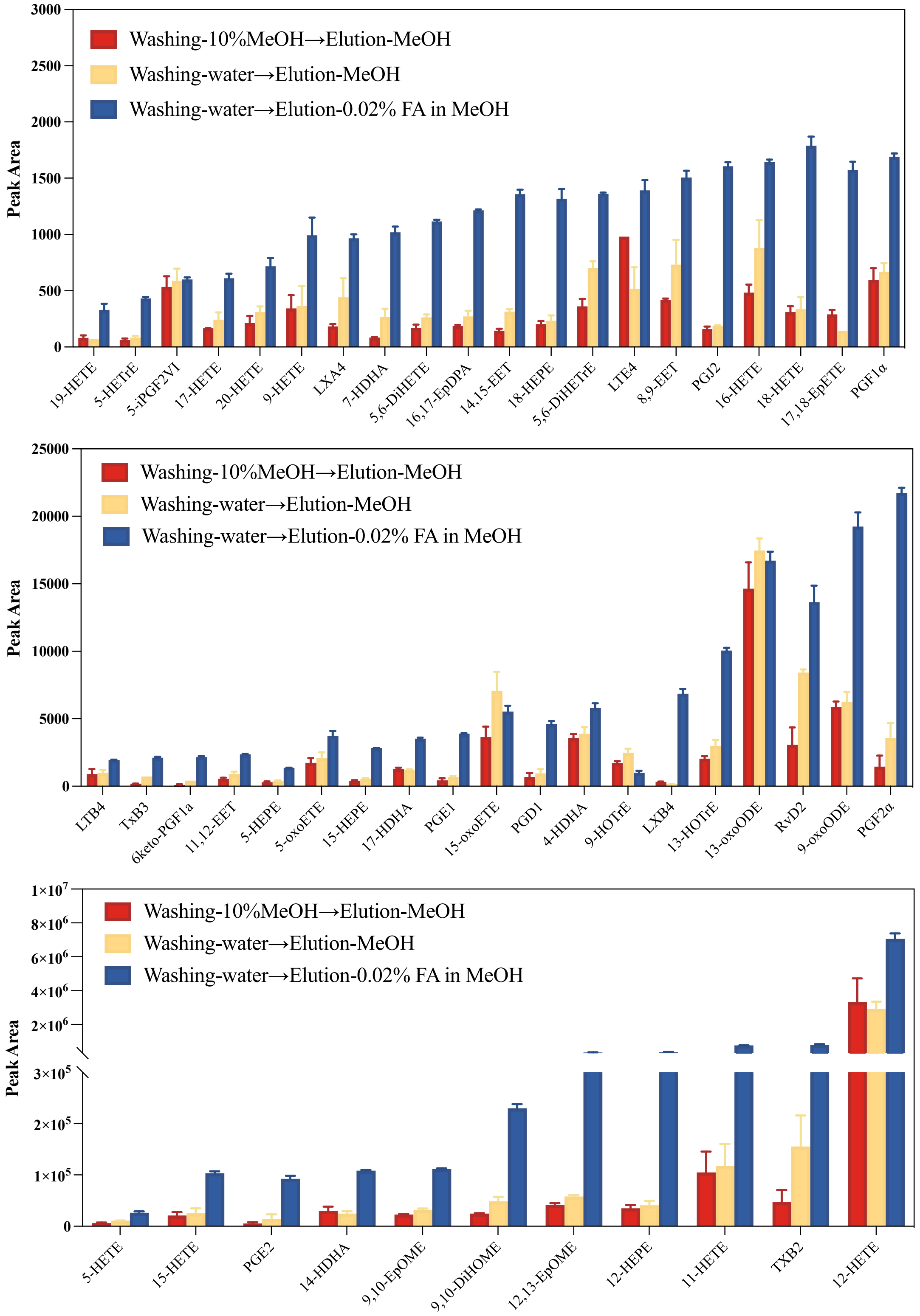

Figure S3. Peak area of oxylipins under different SPE washing and elution solvent conditions.

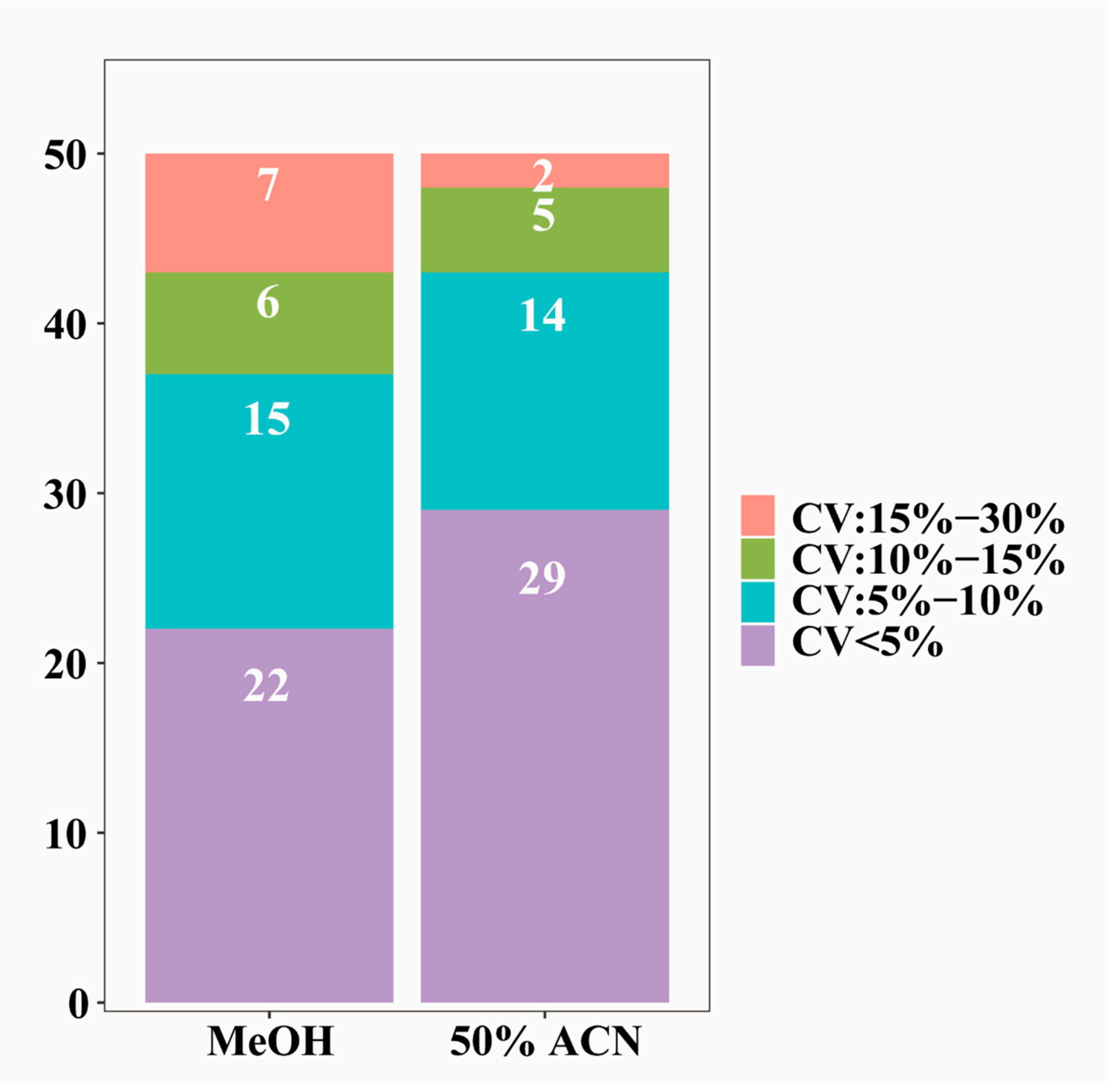

Figure S4. Coefficient of Variation (CV%) of Samples Reconstituted with Different Solvents.

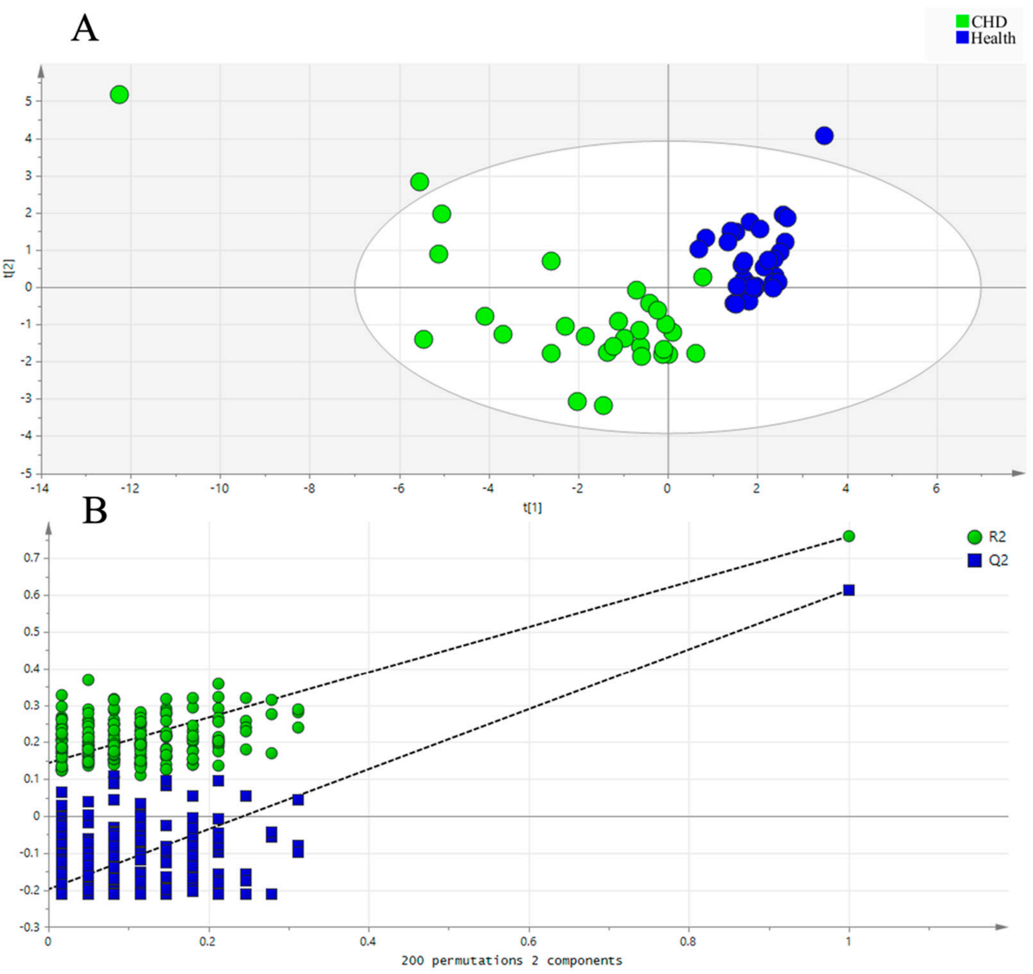

Figure S5. Partial least squares discriminant analysis (PLS-DA) plots (A) and permutation test (n=200) (B) of the PLS-DA model were generated based on serum metabolic profiles detected in negative ion modes between CHD patients and healthy control groups.
